# Genomic and proteomic characterization of multi-drug resistant *Acinetobacter baumannii*

**DOI:** 10.1186/1471-2105-14-S17-A22

**Published:** 2013-10-22

**Authors:** Dana Marshall, Siddharth Pratap, Jianan Dong, Gary Rogers, L Leon Dent

**Affiliations:** 1 Department of Pathology, Anatomy and Cell Biology, Meharry Medical College, Nashville, TN 37208, USA; 2 National Institute for Computational Sciences, Oak Ridge, TN 37831, USA

## Background

Multidrug resistant *Acinetobacter baumanii* (MRAB) is an emerging pathogen that is an important cause of hospital acquired infection and has been shown to increase mortality and length of hospital stay. MRAB is the predominant multidrug resistant bacteria at Nashville General Hospital at Meharry Medical College (NGHM). The goal of this study is to determine the major genomic and proteomics patterns of MRAB at NGHM.

## Materials and methods

The genome of MRAB isolate AB_MMC4 [resistant to over 20 antibiotics, sensitive to tobramycin, possible extended-spectrum beta-lactamase phenotype] was sequenced with a 43 bp single-end run on an Illumina Genome Analyzer II system at the Vanderbilt University Genome Technology Core. Assembly was conducted at the Meharry Medical College Microarray and Bioinformatics Core using BowTie Aligner Software. Gene level annotation was conducted using CuffLINKS software at the University Of Tennessee at Knoxville. A second pass sequencing run was conducted using a 500 bp paired-end strategy on a Roche 454 at the University of Tennessee Center for Environmental BIotechnology. Illumina and 454 hybrid assemblies were done by Cofactor Genmics using Newbler 2.7 on the 454 data and SOAPdenovo 1.05 for assembling the Illumina reads onto the 454 scaffolds. This resulted in 148 contigs and a 3.985 Megabase total length from this clone, yielding high percentage of total genome coverage as MDRAB genomes range in size from 3.5 – 4.3 Megabases.

In order to characterize the antibiotic resistance proteome, AB_MMC4 was grown in plain LB broth or LB broth supplemented with MIC50 concentrations of levofloxacin, tobramycin, gentamicin, cefotaxime and meropenem. Trypsinized peptides were produced from cell lysate and analyzed by LC-MS/MS on a Thermo Finnigan LTQ ion trap mass spectrometer. Tandem mass spectra were acquired using a data dependent scanning mode in which one full MS scan (m/z 200-2000) followed by 5 MS-MS scans and searched against the NCBI MRAB strain ACICU database using MryiMatch and IDPicker software.

## Results

Proteome analysis resulted in 125 high-confidence protein identifications. Antibiotic stress resulted in increased detection of beta-lactamase (cefotaxime is a beta-lactam antibiotic) as well as several proteins associated with oxidative stress that have not previously been described in the context of MRAB resistance mechanisms. These results reinforce the utility of proteomes of antibiotic resistance for MRAB isolates in the identification of potential diagnostic and therapeutic targets, as well as resistance mechanisms, for this emerging pathogen.

